# Biomolecules from Plant Wastes Potentially Relevant in the Management of Irritable Bowel Syndrome and Co-Occurring Symptomatology

**DOI:** 10.3390/molecules27082403

**Published:** 2022-04-08

**Authors:** Ioana-Miruna Balmus, Dana Copolovici, Lucian Copolovici, Alin Ciobica, Dragos Lucian Gorgan

**Affiliations:** 1 Department of Exact Sciences and Natural Sciences, Institute of Interdisciplinary Research, Alexandru Ioan Cuza University of Iasi, Carol I Avenue, no. 11, 700506 Iasi, Romania; ioana.balmus@uaic.ro; 2 Faculty of Food Engineering, Tourism and Environmental Protection, Institute for Research, Development and Innovation in Technical and Natural Sciences, “Aurel Vlaicu” University of Arad, Elena Dragoi St., no. 2, 310330 Arad, Romania; lucian.copolovici@uav.ro; 3 Department of Biology, Faculty of Biology, Alexandru Ioan Cuza University of Iasi, Carol I Avenue, no. 22a, 700506 Iasi, Romania; lucian.gorgan@uaic.ro

**Keywords:** irritable bowel syndrome, plant wastes, fruits, seeds, leaves, pomace, medicinal plant, fibers, fatty acids, hormones, vitamins, enzymes, antioxidants

## Abstract

During and following the processing of a plant’s raw material, considerable amounts are wasted, composted, or redistributed in non-alimentary sectors for further use (for example, some forms of plant waste contribute to biofuel, bioethanol, or biomass production). However, many of these forms of waste still consist of critical bioactive compounds used in the food industry or medicine. Irritable bowel syndrome (IBS) is one of the most common functional gastrointestinal disorders. The primary treatment is based on symptomatology alleviation and controlled dietary management. Thus, this review aimed to describe the possible relevance of molecules residing in plant waste that can be used to manage IBS and co-occurring symptoms. Significant evidence was found that many forms of fruit, vegetable, and medicinal plant waste could be the source of some molecules that could be used to treat or prevent stool consistency and frequency impairments and abdominal pain, these being the main IBS symptoms. While many of these molecules could be recovered from plant waste during or following primary processing, the studies suggested that enriched food could offer efficient valorization and prevent further changes in properties or stability. In this way, root, stem, straw, leaf, fruit, and vegetable pomaces were found to consist of biomolecules that could modulate intestinal permeability, pain perception, and overall gastrointestinal digestive processes.

## 1. Introduction

Plant wastes are common end-products of the industries developed by humankind across the centuries. The food industry, animal husbandry, pharmaceutical industry, and clothing industry use plants as raw materials in obtaining different products for whom use is essential. However, substantial wastes resulting from fruit, vegetable, and medicinal plant processing could often pose an important threat to the environment and lead to economic imbalance. For instance, some fruits, leaves, straws, or even roots are wasted in proportions varying from 10 to 65% of the whole plant’s weight [1].

It is generally accepted that many forms of plant processing waste can be reutilized, as some bioactive components could still be retained. In this way, it was shown that plant waste could be valorized in obtaining biofuels, bio-fertilizers, and bioethanol. Additionally, it was shown that plant waste could be used in the food industry to improve nutritional food quality or design foods that address some special dietary needs of individuals [2]. Thus, functional foods could be an essential component of the human diet and health. In this way, since much plant waste originates from the food processing industry, and since studies have shown that fruit and vegetable waste is still a rich source of nutrients and bioactive molecules [3], it could be suggested that this waste could be further used in alimentation by further processing. Therefore, it could be useful to find ways to include processed plant waste in functional foods, which are enriched dietary products of significant use in health and diseases [4,5], such as digestive disorders, including the most common chronic functional gastrointestinal disorder, irritable bowel syndrome [6,7].

**Figure 1:**
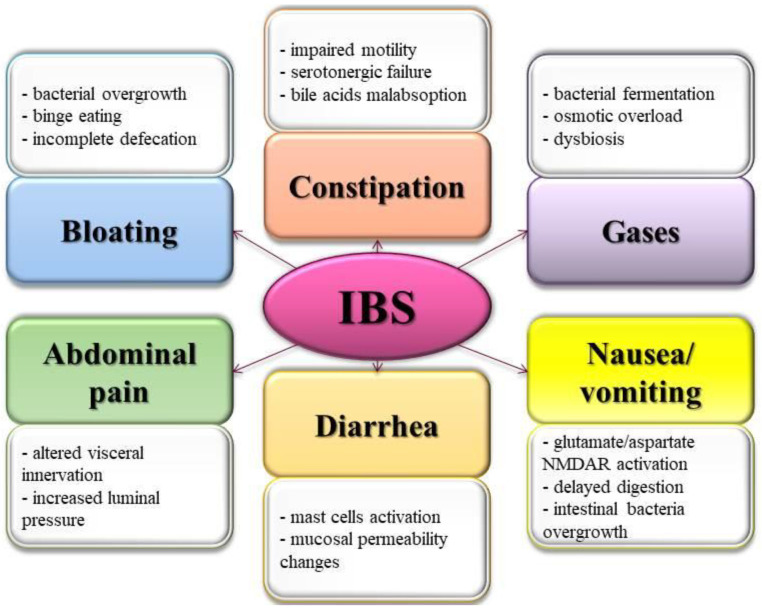
Pathological mechanisms of the main IBS symptomatology (according to ROME IV Diagnostic Criteria for functional gastrointestinal disorders) (NMDAR: NMDA receptor) [10,20,21,22,23,24,25,26,27].

Irritable bowel syndrome currently affects one in every five individuals [8,9]. Despite the patients describing the symptomatology as extremely disturbing, no significant tissue and molecular changes or damage have been reported [10]. Thus, IBS diagnosis is almost exclusively based on clinical symptomatology (Figure 1). In this context, studies have failed to identify an apparent cause of IBS occurrence. Still, the evidence has shown that IBS could result from a complex interaction between the gastroenterological, neurological, and molecular components observed in its pathology, as previously described [11]. Additionally, several key risks and promoting factors were established, one of which is the modern Western diet [12].

While it was shown that an imbalanced and nutritionally deficient diet could promote, maintain, and worsen IBS symptoms [13], several studies have documented significant improvement in IBS symptomatology directly correlated with changes in dietary habits [14,15,16,17,18]. The FODMAP diet is just one of the current food plans used to alleviate IBS gastroenterological symptoms by assessing each aliment’s implications following the IBS patient’s tolerance to it or its benefits [19].

Considering these aspects, this review aims to identify and describe the potential beneficial components of plant processing waste in IBS management and propose possible uses for this waste according to recent studies on food processing, industry, and the nutritional values of fruit- and vegetable-waste-enriched food products.

## 2. Dietary Fibers

While dietary fibers’ exact definitions and classifications are still incomplete, they were initially considered bulk material in vegetable food sources. Their properties enable them to be of extreme use in gastrointestinal tract health. In this way, the soluble dietary fibers (pectin, gums, and mucilages) were demonstrated to interact with some of the molecules which contribute to digestive processes, such as bile acids, gastric hydrochloric acid, and heavy metals, while the insoluble dietary fibers (cellulose, hemicelluloses, and lignin) contribute to stool bulk weight and intestinal peristalsis (thus shortening the GIT transit time and preventing the long interaction of toxic stool components with the intestinal lining) [2,28]. Furthermore, it was demonstrated that the gut microflora directly benefits from the dietary fiber intake, as the soluble fraction undergoes bacterial fermentation. In contrast, the insoluble fiber prevents putrefactive bacteria growth and stimulates gut microbiota development and diversity [2]. Thus, it was shown that dietary fibers exhibit multiple effects, including the prevention of several critical modern diseases such as metabolic diseases, cardiovascular disorders, and gastrointestinal tract malignancies [29].

In this context, the fact that the management of IBS includes dietary control offers sufficient background to consider diet one of the best ways to manage IBS. It was previously shown that food could be the primary method through which IBS symptom burden could be modulated [16]. In this way, the encouragement to increase the dietary fiber intake was an important strategy to improve intestinal transit in constipation-predominant IBS and mixed IBS patients [30]. Additionally, since many diarrhea-predominant IBS cases are associated with bile acid malabsorption [31], increased dietary fiber intake could lead to bile acid secretion modulation and the binding of bile acids molecules in the colon to ensure their passage and thus prevent reabsorption [31,32]. Furthermore, as previous studies have shown that plant waste could be affected by further processing, the minimal transformation of plant material could be undergone by endeavors to add in enriched foods [5,33].

Now, quantity is always significant, but more important is quality. It is relevant to mention that many studies suggest that almost all vegetable food source waste could offer good quality dietary fibers. Hussain et al. [1] extensively discussed the dietary fiber content of different plants, often underutilized and wasted, and described them considering their sources. Starting with fruit juice by-products and ending with the precooked meals industry, the amount of raw material wasted could, in some cases, reach as high as 50% (for *Citrus x sinensis* and *Mangifera indica* fruits). It was suggested that the pomace originating from juice manufacturing could be rich in dietary fibers. The most abundant pomace, resulting from *Citrus* fruit processing, was demonstrated to be extremely rich in pectin [34], which could offer additional protection for the intestinal cells by strengthening the mucous layer, preserving the epithelial integrity, modulating the innate intestinal immune system, and reducing coliform adhesion to colon mucosa [35,36]. Rodriguez et al. [34] mentioned *Psidium guajava* and *Mangifera indica* fruit pomaces resulting from juice manufacturing as a possible dietary fiber source. Additionally, *Malus domestica* and *Pyrus communis* fruit pomaces were described as rich in pectin content [2].

Regarding the soluble fiber content, it was reported that *Ribes nigrum* and *Vitis vinifera* fruits are particularly rich in this type of fiber [27]. Nawirska and Uklanska [29] showed that several *Malus domestica*, *Aronia arbutifolia*, *Ribes nigrum*, *Fragaria × ananassa* fruits and *Brassica oleracea (var. capitata f. rubra*) and *Daucus carota* pomaces are high in neutral and acidic dietary fiber content. The highest dietary fibers were observed in *Aronia arbutifolia* and *Ribes nigrum* fruit pomaces, while the lowest content was observed in the *Malus domestica* fruit pomace. Considering that each dietary fraction could exhibit a different role in IBS management, significant differences between soluble and insoluble fibers were noted. In this way, *Aronia arbutifolia* and *Ribes nigrum* fruit pomaces were rich in insoluble dietary fibers. In contrast, *Daucus carota* roots and *Malus domestica* fruit pomaces were rich in soluble dietary fibers [28]. Considering the high importance of soluble dietary fibers in IBS, Alba et al. [28] discussed that *Ribes nigrum* and *Vitis vinifera* fruits could be abundant sources of this fiber. Moreover, it was suggested that grape pomace could be an essential component of the human diet due to other constituents besides dietary fibers, anthocyanins, and ellagitannins, as Alba et al. [28] described.

Other plant processing wastes that could be successfully used to produce dietary fiber-enriched foods are *Asparagus officinalis* spears, which include almost 50% of the harvested part of the plant that are lost mainly during the preparation process [34]. Studies have shown that *Asparagus officinalis* is rich in soluble and insoluble dietary fibers and phenolic compounds, which could partly motivate its increased antioxidant activity [37]. Furthermore, the peels of *Musa* sp., *Mangifera indica*, *Psidium guajava*, *Colocasia esculenta*, *Averrhoa carambola*, *Pouteria sapota*, *Manilkara zapota*, and other exotic fruits were described to be ideal as dietary fiber sources [28]. Additionally, the seeds of fruits could be used to produce dietary fiber and other potentially active by-product extracts. Thus, the seeds of apples and grapes were demonstrated to consist of soluble and insoluble fibers, according to Carle et al.’s patent [38] and Schieber et al. [39], which are both cited by [27], who argue the importance of dietary fibers alongside the antioxidant derivates originating in the mentioned fruits’ wastes. Similar properties and contents were reviewed for *Actinidia deliciosa* and *Olea europaea* fruit waste and *Ananas comosus* peels.

Considering their properties, how the discussed plant waste dietary fiber extracts could be used were previously discussed. Thus, many dietary fiber extracts could be used as soluble powders, and those already available for commercial use (i.e., Nestle’s Optifibre product, a pea fiber powder soluble formula for nutritional supplementation) could be added to different food products. In this way, bread (*Triticum aestivum* bran, *Solanum tuberosum* tubercle peel), biscuits and cookies (*Oryza sativa* bran, *Musa* sp. fruit waste flour, *Malus domestica* and *Citrus sp* fruit pomaces, *Daucus carota* root pomace, *Solanum tuberosum* tubercle peels, and *Pisum sativum* seed peels), yogurts (*Avena sativa* bran, *Citrus* sp. peel extract, *Bambusa vulgaris*, *Malus domestica*, *Phoenix dactylifera* fibers), cheese (waste products rich in gums, pectin, and inulin), ice cream (*Ananas comosus* waste products), sweets (marmalades, chocolate), meat products (*Glycine max*, *Triticum aestivum Zea mays*, *Oryza sativa* brans, *Ananas comosus* peel, *Psyllium* mucilloid, *Pisum sativum* and *Cicer arietinum* hulls, apple, and Lagenaria siceraria pulp) were demonstrated to be efficient and nutritionally improved products in which dietary fibers originating from plant waste fit perfectly and offered health benefits [27,38,39,40].

*Solanum tuberosum* tubercle peels (up to 68% of the total mass of the potato) could be used in different bakery products considering their dietary fiber content and properties [41]. Potato peels consist of large amounts of dietary fibers, and their properties could enhance the quality and nutritional value of the products. Similarly, *Pisum sativum*, *Lens lenticularis*, and *Phaseolus vulgaris* seed peels could also be used as a source of dietary fiber that could play an essential role in alleviating the digestive symptoms of IBS (i.e., constipation, diarrhea). *Persea americana* fruit peels and seeds were also a good source of dietary fiber, protein, and micronutrients [42] which can be used in the food industry or functional foods associated with IBS needs.

However, Capili et al. [31] suggested that some dietary fiber sources could pose severe problems due to the increased content of fermentative fibers, the rapid fermentation of which could lead to bloating and abdominal discomfort. Thus, several vegetables (*Brassica oleracea*, *Allium cepa* stems, *Pisum sativum/Phaseolus vulgaris*, *Lens lenticularis* seeds, *Solanum tuberosum* tubercles), grains, and fruits (*Musa* sp. and some *Citrus* varieties) [30,42], could be the source of such dietary fibers, which are to be avoided by some IBS patients. Additionally, several studies evaluated the effect of dietary fiber processing (extraction methods and further processing to final products) and suggested that it could influence their total content and quality. Thus, it was shown that enzymatic processing of the raw material could lead to an essential decrease in this fraction, as Nawirska and Uklanska [29] discussed for *Daucus carota* root pomace.

## 3. Lipids

Many forms of lipid-rich plant waste are currently used in the biofuel industry. In the oil industries, the consistent amounts of plant residues following edible oil extraction, generally achieved by processing the seeds through mechanical pressing, results in oil cakes. Due to their increased fat and other oily components, they pose an essential threat to the environment, with their disposal often leading to pollution [29]. Thus, resources such as *Elaeis guineensis*, *Cocos nucifera*, and *Olea europaea* fruits, *Helianthus annuus*, *Brassica napus* (canola), *Sesamum indicum*, *Camelina sativa*, and *Sinapis alba* seeds, and others, are currently used to produce edible oils, while the residues are wasted [43]. However, their further potential could include valorization since the oil cakes still consist of significant nutritional molecules and are a relevant source of proteins, fibers, microelements, lipids, and other active molecules (polyphenols, carotenoids, anthocyanins, etc.) [43].

However, the overall effects of dietary lipids on the IBS-affected gastrointestinal tract are somewhat controversial. Since lipid metabolism was described as impaired in IBS, the high intake of dietary lipids could lead to bloating, gas retention, and flatus [44]. As previously discussed, a lipid-rich diet should always be replaced by a dietary fiber-rich diet to prevent the premises that lead to constipation [2]. Additionally, the impaired lipid metabolism in IBS was suggested to be the source of small intestine motor dysfunction [45], which further impaired gastrointestinal transit and predisposed to hypersensitivity to distension (often the leading cause of pain and urgency) [46]. The correlation between increased lipid consumption and abdominal pain was also demonstrated by Simrén et al. [47], who observed that a colonic lipid infusion leads to increased colonic sensitivity and altered viscerosomatic perception. Thus, a lipid-rich meal would not be recommended to IBS patients.

Despite this, several classes of lipids are of extreme use in human health and gastrointestinal tract active function. For instance, Chua et al. [48] suggested that the decrease of fatty acids in the plasma of IBS patients could be correlated to characteristic symptoms of IBS. Moreover, Clarke et al. [49] showed that an imbalance between pro-inflammatory and anti-inflammatory fatty acid levels could partly explain the inflammation mechanism in IBS pathogenesis. In this way, fatty acid supplementation in IBS patients’ diets could improve the intestinal inflammatory response. Michalak et al. [50] presented the hypothesis that lipid profile and gut microbiota are interrelated in IBS, and palmitoleic acid, docosahexaenoic acid, or propionate could be involved in the occurrence of IBS symptoms. In contrast, other polyunsaturated fatty acids could act as visceral sensitivity inhibitors. However, the authors pointed out that polyunsaturated fatty acid supplementation’s clinical effect was not yet evaluated.

Recent studies showed that just a few types of plant waste could be a relevant source of lipids and free fatty acids used in the human diet. An important source of lipids could be medicinal plant wastes, as Khomova et al. [51] described in 1996. The authors did not discuss the purpose of these waste-originating lipids, but medicinal plant waste could be considered a safe source for human diets. However, some coercion could be formulated due to the processing methods applied in extracting the active principles from the medicinal plants, often involving the use of possibly harmful solvents, such as organic solvents, acids, and alcohols [52]. In this way, Khomova et al. [51] reported that resinous, liquid and solid residues of medicinal plant preparation processes could have consisted of significant amounts of lipids and fatty acids, as high as 97–98% of the waste weight: wax esters (consisting of fatty alcohols potentially hydrolyzed to free fatty acids, which when originating from marine animals could prevent constipation and modulate inflammation [53]), ester sterols (possessing the potential to modulate cholesterol metabolism [54]) and triterpenols (derivates of which exhibit antioxidant and anti-inflammatory properties [55]).

Other ways of obtaining good quality edible lipids include using plant waste as a substrate for lipid-producing microorganisms and genetically engineering plants. In this way, a wide range of plant waste is currently used as substrates for lipid-rich biomass production: cereal grains waste originating from *Triticum aestivum*, *Hordeum vulgare*, *Oryza sativa*, and *Zea mays* plant processing (bran, straws, combs) and legume seed waste (mainly lignocellulose sources) can be used for fermentative bacteria and fungi growth [44]. Two of the most common fermentation food products originating from plant waste are oncom and tempeh, both of which are obtained by the fermentation of *Arachis hypogaea*, *Sesamum indicum* seed cakes, *Vigna radiata* wastes, and *Glycine max* wastes. The most important aspect of discussing these waste valorization products is the potential benefits of alleviating IBS symptoms. Despite the increased content of raffinose, stachyose, and phytic acid (capable of producing bloating and gas retention), IBS patients could trigger supplemental gastrointestinal symptoms. These side effects could be successfully overcome if *Rhizopus oligosporus* and *Neurospora sitophyta* carry out the fermentation, both being able to hydrolyze phytic acid and the mentioned saccharides, thus eliminating the risk for flatulence development [56]. Moreover, it was shown that oncom could promote gut microbiome growth and development since both of them used fermentative species are considered probiotics [57]. Furthermore, tempeh was revealed to be a valuable source of good quality proteins, especially when prepared from soy milk waste, and an excellent probiotic source [44,58].

Non-edible oil waste could also be valorized in the food industry by being used as a substrate for yeasts (i.e., *Yarrowia lipolytica*) that produce lipid-rich biomass and an important lipid metabolism enzyme (lipase) [59]. Zinjarde et al. [60] documented the use of *Yarrowia lipolytica* in meat and dairy products. They observed its lipolysis activity and its capacity to produce proteases and esterases, which promote maturation processes. However, protease-rich sources could lead to the inflammatory response [61], and thus it could be considered an IBS trigger. Furthermore, according to Park et al. [62], *Yarrowia lipolytica* yeast could be genetically engineered to produce heptadecenoic acid, which was demonstrated to increase intestinal motility in a rat model of diarrhea in neo-maternal separation by modulating the frequency of colonic muscle contractions [63].

Regarding transgenic plants that exhibit increased contents of polyunsaturated fatty acids, Khan et al. [64] reviewed several species which were exceptionally engineered to produce omega-3 fatty acids. Thus, transgenic *Camelina sativa*, *Glycine max*, *Brassica carinata*, and *Brassica nigra* seeds could all produce 15 to 25% eicosapentaenoic acid and docosahexaenoic acid [64], both helpful in the management of IBS symptomatology, specifically in gastrointestinal and mood impairments [48,65,66].

## 4. Vitamins and Minerals

**Figure 2:**
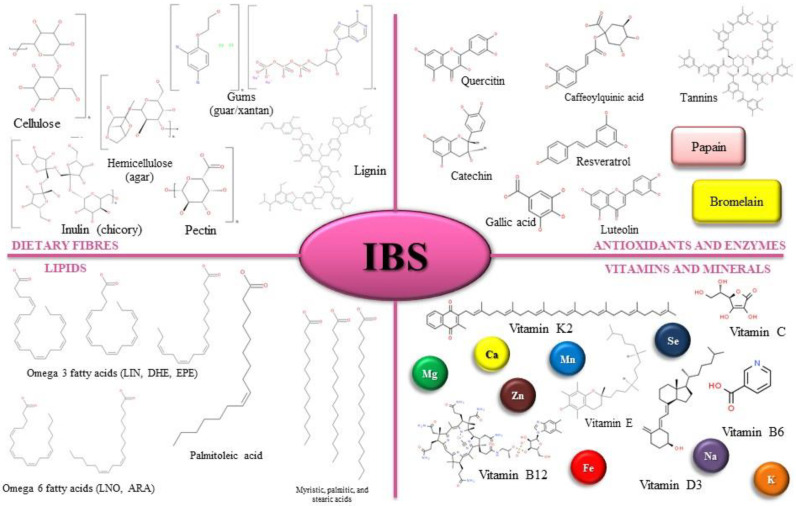
Biomolecules that are potentially relevant to IBS symptomatology management (molecular structures added using BIOVIA Draw 19 software, 2019 by San Diego: Dassault Systèmes and IUPAC names available on PubChem database).

The importance of micronutrients in the diet of IBS patients was discussed by several studies, which suggested that supplementation with some vitamins could be of extreme use in the alleviation of IBS symptoms. Considering the gastrointestinal impairments occurring in IBS and the tendency of the patients to avoid consuming certain aliments and foods, it was also discussed that some vitamins and minerals could be deficient or lacking in IBS patients, which could lead to further impairments. Thus, it was shown that IBS patients often lack vitamin D and minerals, such as zinc, iron, and magnesium [67,68]. Therefore, it could be suggested that different nutrient malabsorption could cause these deficiencies, which form the premises for other comorbid impairments, such as dyspepsia, colitis, IBD, diabetes, anxiety, depression, and chronic pain [31,69,70]. Moreover, a recent study showed that vitamin D supplementation in IBS patients could improve symptomatology [71]. At the same time, Davis [72] reported that practitioners also prescribed vitamins and minerals to manage IBS (vitamins B12, C, D3, and K2, and magnesium) (Figure 2).

Furthermore, Jalili et al. [73] showed that *Glycine max* seed isoflavone and cholecalciferol (vitamin D3) administration to IBS patients could significantly improve gastrointestinal symptoms by reducing inflammation and gut permeability. B complex vitamin deficiency was documented in IBS patients in correlation to gastrointestinal symptomatology and comorbid psychiatric manifestations [74,75,76]. Thus, the importance of vitamins and minerals in the management of IBS is evident. In this way, it was shown that many forms of plant waste are still rich in vitamins and minerals and could successfully be considered to be sources for further processing.

Khattak and Rahman [77] reported that the peels of many vegetables consist of increased amounts of vitamins and minerals. By evaluating the peel samples from *Beta vulgaris*, *Brassica rapa*, *Daucus carota*, and *Raphanus sativus* roots, *Zingiber officinale* rhizomes, and *Ipomoea batatas* and *Solanum tuberosum* tubercles, increased levels of vitamin C (*Raphanus sativus*, *Brassica oleracea var. botrytis*), riboflavin (*Ipomoea batatas*), niacin (*Brassica rapa*), and thiamine (*Solanum tuberosum*) were reported. Furthermore, they compared their results with other studies. They reported that similar levels of the mentioned vitamins were observed in *Solanum melongena*, *Abelmoschus esculentus*, *Capsicum annuum*, *Brassica oleracea var. botrytis*, *Solanum lycopersicum*, and *Lagenaria siceraria* fruits, and *Spinacia oleracea*, *Brassica oleracea*, and *Lactuca sativa* leaves [78,79,80]. Vitamin C was also reported in *Sambucus nigra*, *Sorbus aucuparia*, and *Rosa canina* fruit pomace [81]. Moreover, other possible sources of fruit waste, such as *Psidium guajava*, *Carica papaya*, *Prunus domestica*, *Prunus persica var. nucipersica*, and *Vitis vinifera* fruit peels, were evaluated for their vitamin content. *Spondias tuberosa* fruit pulp contains increased amounts of vitamin C and B complex vitamins [78,82,83].

Some of the B complex vitamins, as well as vitamin C and E, were reported in *Musa* sp. fruit peels, which are more than one-third of the fruit and are often wasted or composted [42,84]. Moreover, Amini Khoozani et al. [85] demonstrated that unripe banana peel flour could exhibit beneficial effects on colon health due to the increased content of resistant starch. In contrast, ripened banana peel flour could improve digestion due to its starch and protein contents. Additionally, Pathak et al. [86] reviewed the potential of *Carica papaya* fruit peels to be used in medicinal applications, and considering the rich range of minerals, vitamins, phenolic antioxidants, and dietary fibers, they reported that papaya peels could successfully be used in food, cosmetic, and pharmaceutic industries, as they have the potential to exert anti-inflammatory effects, prevent aging and colon cancer, and facilitate pain relief, muscular relaxation, and smooth muscular contraction. Thus, papaya peels could be considered to be of possible relevance in the alleviation of IBS symptomatology.

Another essential vitamin for the integrity and health of the gastrointestinal tract is vitamin E, which is present in a vital amount in *Vitis vinifera* seeds [42]. At the same time, vitamin E was demonstrated to exhibit modulatory effects on the oxidative stress balance and intestinal epithelial barrier function [87,88] and improve and protect gut microbiota [89] in various animal models of relevant gastrointestinal impairments for IBS pathological mechanisms. Alongside vitamin C, vitamin E was identified in the peels and seeds of *Mangifera indica* fruits, which can successfully be processed into biscuits and gelatins [42].

In a close relationship with vitamins, metal microelements are also important in human health and nutrition. Thus, it was shown that trace elements such as magnesium, zinc, copper, and iron are important modulators of the innate immune system [90]. Previous studies on trace biometals showed deficiencies of iron [91], zinc [68], magnesium [92], selenium [93], calcium, phosphorus [94], and potassium [95] in IBS or IBS-associated pathologies. Similarly, Hujoel [68] reported an imbalance in the copper-zinc ratio in IBS patients and pointed out their implication in the brain-gut axis and gastrointestinal barrier functions. There is no clear explanation of the correlation between micronutrient deficiency and IBS. Still, it was repeatedly suggested that the nutritional limitations occurring in IBS patients by frequently avoiding certain types of foods could lead to micronutrient deficiencies and other associated disorders such as iron deficiency anemia [96], migraine [97], depression, and anxiety [67,98].

Considering these aspects, different formulations or functional foods obtained using plant waste as raw material could bring the discussed micronutrients into the IBS diet. For instance, Krupa-Kozak et al. [99] examined the use of *Fagopyrum esculentum* grain flour in gluten-free bread. Knowing that IBS patients are often challenged by digestive sensitivity to gluten [100], it could be of increased interest to replace regular bread with gluten-free bread, as proposed by Krupa-Kozak et al. [99]. It was shown that the benefits of this formulation extends the visual quality of bread and micronutrient composition, as iron, zinc, copper, and manganese contents were directly dependent on the supplementation of *Fagopyrum esculentum* grain flour. *Fagopyrum esculentum* processing waste (straws and husk) are currently used in bioethanol production [101] or as sorption materials for the removal of pollutants from aqueous media production, i.e., heavy metals [102], but their use could be extended as microelement sources, as Zemnukhova et al. [103] reported high contents of potassium, sodium, calcium, magnesium, zinc, manganese, iron, copper, cobalt, and iodine in buckwheat straws and husks.

Moreover, Khattak and Rahman [77] showed that different forms of vegetable peel waste such as *Beta vulgaris*, *Brassica rapa*, *Daucus carota*, *Raphanus sativus* roots, *Zingiber officinale* rhizomes and *Solanum tuberosum* tubercles carry increased levels of microelements, including sodium, calcium, magnesium, iron, zinc, potassium, and phosphorus, which could be of nutritional interest in the human diet. Additionally, the authors [77] reviewed other possible sources of microelements: *Punica granatum* fruit peel powder (potassium, sodium, iron, manganese, and zinc contents) [104] and leafy vegetables (calcium, zinc, manganese, iron, and magnesium) [105].

## 5. Digestive Enzymes

Digestive enzyme supplementation is a current treatment for gastrointestinal symptoms of IBS based on the administration of digestive enzymes in different formulations to facilitate or modulate digestion and intestinal motility in IBS patients [106,107]. In this way, Spagnuolo et al. [108] showed that digestive enzyme supplementation alongside beta-glucan and inositol could lead to significant improvements in abdominal pain, bloating, and flatulence. Similarly, Graham et al. [107] suggested the oral administration of digestive enzymes and modulators such as protease, amylase, pancreatin, bile salts, betaine hydrochloride, hemicellulase, and cellulase could provide significant benefits in the management of functional digestive disorders. Furthermore, the administration of pancreatic enzymes in diarrhea-predominant IBS patients significantly improved stool consistency and abdominal pain. At the same time, the authors thoroughly documented possible pancreatic insufficiency in diarrhea-predominant IBS [107].

The best-known example is *Carica papaya* fruit peel extract, of which the principal constituent, papain, is considered relevant in alleviating digestive symptoms occurring in IBS [108]. Thus, the fact that papaya fruit preparations exhibit properties such as histaminic receptor modulation (involved in pain sensation, but also gastric acid synthesis) [109] and that several studies report significant improvements in diarrhea, constipation, flatulence, discomfort, and rectal sensibility to distension [110,111], *Carica papaya* fruit wastes could be considered in being valorized with the purpose to manage IBS symptoms.

Additionally, similar effects were reported for bromelain, naturally found in the fruits and the stems of *Ananas comosus* [112]. Bromelain was also demonstrated to modulate the intestinal inflammatory and immune response [113], suggesting a possible relevance in post-infectious IBS. Due to its active properties in reducing bloating, gas, pain, and inflammation, pineapple peels and pomaces consisting of increased amounts of bromelain could be taken into consideration to be further valorized for IBS management in formulating supplements or by being added to functional foods, as previously discussed [113,114].

## 6. Antioxidants

A recent report showed that oxidative stress is an essential component of IBS’s complex and multifactorial pathophysiology [115]. Moreover, this aspect was previously addressed in animal model studies showing that oxidative balance in IBS could be significant in both gastrointestinal and neurological components, involving the gut-brain axis and the stress axis [9]. In this way, one could suggest that antioxidant therapies are significant adjuvants in IBS management and control. Mete et al. [116] even proposed an extensive discussion of the possible relevance of oxidative and nitrosative stress in the etiology of IBS. Additionally, concomitant with Khan et al. [117], who reviewed the symptomatology relief which can be achieved by the administration of different plant extracts, the evidence of the possible modulatory role of oxidative stress in IBD stands [118], which could be similarly improved by antioxidant administration, as reviewed by Moura et al. [119].

Considering the new guidelines in IBS diagnosis and treatments, an antioxidant-rich diet is encouraged due to the significant improvement seen with antioxidant administration. In this context, there are plenty of antioxidant sources that can provide quality components to the human diet, including plant waste from the food industry. In this way, our group previously studied the antioxidant potential of *Camelina sativa*. We showed that the oil obtained from the *Camelina* seeds could improve antioxidant enzyme activity and prevent lipid peroxidation in a rat model of stress exposure (IBS model) [120]. Moreover, Mierina et al. [121] showed that the antioxidant potential is also preserved by the seed cakes, not only the oil. Thus, pressed cakes could successfully be an antioxidant source to incorporate into functional foods.

Additionally, it was previously shown that *Vitis vinifera* fruit pomace could exhibit antidepressant and anxiolytic properties, which could also help manage IBS, as depression and anxiety are burdensome components of IBS comorbid impairments [9,122]. Based on the previous description of grapes’ pomace chemical composition [123], consisting of anthocyanins, alongside an actual number of dietary fibers, pectin, proteins, minerals, and vitamin C, it was shown that grape pomace could significantly improve IBS symptomatology related to mood and memory in a rodent model. Anthocyanins and their antioxidant potential were recently described by Khoo et al. [124] and Tena et al. [125]. Significant amounts of anthocyanins were found in other fruit and vegetable pomaces such as *Malus domestica*, *Aronia melanocarpa*, *Daucus carota* ssp. *sativus var. atrorubens* [29], and *Citrus* sp. wastes [41]. An extensive analysis of the antioxidant content of fruits and vegetable wastes was carried out by Deng et al. [3].

Another essential antioxidant class that can be found in fruits and vegetables is polyphenols. Eskicioglu et al. [126] reviewed the content of polyphenols and similar compounds, which can exhibit antioxidant capacity in the presence of dietary fibers (thus, antioxidant dietary fibers). Polyphenols, flavonols, carotenoids, and other antioxidants were reported in fruit and vegetable pomaces and wastes, such as *Vitis vinifera*, *Malus domestica*, *Euterpe oleracea*, *Opuntia humifusa*, *Psidium guajava*, *Cucumis melo*, *Passiflora edulis*, *Citrus limetta*, *Ananas comosus*, *Solanum lycopersicum* fruits, *Daucus carota* roots, *Brassica oleracea* leaves, and the seeds wastes of *Theobroma cacao* and *Coffea* sp. [127,128]. Despite many examples, the berries remain the most significant source of antioxidant compounds (*Aronia melanocarpa*, *Ribes nigrum*, *Rosa canina*, *Sambucus nigra*, *Sorbus aucuparia*, *Morus alba*, *Morus nigra*) [29,126,128,129]. Still, the relevant compounds were also present in less mentioned plant wastes, such as *Punica granatum* fruit peels [3] and *Castanea sativa* seed epicarps [130].

Regarding the industrial ways in which the mentioned pomaces could be valorized to bring significant nutritional value and symptomatologic relief, Yu [131] reported the nutritional and sensory quality of bread in which *Vitis vinifera* fruit pomaces were incorporated. Similarly, Ajila et al. [132,133] described the antioxidant potential of *Mangifera indica* fruit peel powder when added to biscuits and macaroni. Additionally, it was recently reported that fruit pomaces could significantly improve the nutritional value of biscuits, cookies, cakes, and bread products and exhibit antioxidant potential [81,134,135,136,137,138,139]. *Armoracia rusticana* [138], *Daucus carota* [127], and *Beta vulgaris* roots [140] were suggested as possible sources of antioxidant components that could be of use in functional foods.

Antioxidants could also be found in medicinal plant wastes originating from essential oils or tea/juice concentrate extractions. Thus, it was previously shown that waste resulting from aromatic plant distillation (*Thymus vulgaris* and *Origanum vulgare*) could be a possible source of antioxidants and phenolic compounds [141]. Similarly, Yantcheva et al. [142] reported that *Matricaria chamomilla* waste could be a significant source of antioxidants. These forms of plant waste could also be valorized in food products. In this context, Ning et al. [143] reported that *Camellia sinensis* waste could significantly improve the quality of bread products by adding antioxidant potential.

However, the antioxidant capacity decreases with increased processing steps until the end product [138]. Thus, it should be recommended that fruit and vegetable pomaces and wastes be valorized using as few processing techniques as possible to preserve the active principles. Despite their increased potential for further utilization, the need for fewer processing techniques required to preserve many of the active compounds could further impose some limitations. In this way, the contamination of vegetal food sources with heavy metals and microbial agents was previously shown [144,145,146]; thus, it must be thoroughly assessed in further strategies of plant waste valorization.

## 7. Conclusions

Significant evidence has shown that many forms of fruit, vegetable, and medicinal plant waste could be a source of critical bioactive molecules that could be implicated in IBS treatment or prevention: dietary fibers, lipids, vitamins, minerals, enzymes, and, last but not least, antioxidants, many of which could be recovered during or following processing without interfering with or hindering the primary technological processes. Root, stem, straw, leaf, fruit, and vegetable pomaces were rich in the mentioned biomolecules and thus relevant in further strategies of IBS management.

## Data Availability

Not applicable.
